# Optimization of Injection Molding Processing Parameters for Thin-Walled Plastic Parts Manufactured for the Automotive Industry

**DOI:** 10.3390/polym18010091

**Published:** 2025-12-28

**Authors:** Nedime Ozdemir Potuk, Mustafa Oksuz, Aysun Ekinci, Murat Ates, Ismail Aydin

**Affiliations:** 1 Isringhausen Seat Supplier, Demirtas Dumlupınar OSB, 16245 Bursa, Turkey; nozdemir@isri.com.tr; 2 Faculty of Engineering and Architecture, Recep Tayyip Erdogan University, Zihni Derin Campus, 53100 Rize, Turkey; 3 Department of Polymer Materials Engineering, Faculty of Engineering, Yalova University, 77200 Yalova, Turkey; 4 Department of Chemistry, Faculty of Science, Nigde Omer Halisdemir University, 51100 Nigde, Turkey; aysun.ekinci@yalova.edu.tr; 5 Department of Materials and Materials Processing Technologies, Yalova Vocational School, Yalova University, 77200 Yalova, Turkey; 6 Department of Chemistry, Faculty of Arts and Sciences, Tekirdag Namik Kemal University, Degirmenalti Campus, 59030 Tekirdag, Turkey; mates@nku.edu.tr; 7 Nanochem Polymer Energy Company, Silahtaraga Mah., University 1st Street, Number 13/1 Z242/1, 59860 Tekirdag, Turkey; 8 Rheology Laboratory, Department of Chemical Engineering, Faculty of Engineering, Istanbul University-Cerrahpasa, 34320 Istanbul, Turkey; i.aydin@iuc.edu.tr

**Keywords:** thin-walled, automotive, plastic, optimization, design of experiments

## Abstract

The fabrication of thin-walled plastic parts has potential in the automotive industry in terms of sustainability and circular economy targets to decrease any harmful effects on the ecosystems, cost and performance. Injection molding of thin-walled automotive parts is more complex in terms of processing defects compared to traditional plastic parts. Optimization of processing parameters is of critical importance to solving problems and defects in the production of thin-walled parts. In this study, the flow length and weight of thin-walled spiral parts (with wall thicknesses of 0.50, 1.50, 2.70 and 3.00 mm) were investigated with theoretical and experimental studies. The theoretical flow length and weight of the thin-walled spiral parts were determined by Moldflow analysis according to the pressure and wall thickness. The correlation graph between theoretical results and experimental measurements was obtained. When the wall thickness of the thin-walled spiral parts increased, the flow length of the thin-walled spiral parts increased. As a result, it was found that the thin-walled spiral part mold could not be filled for wall thicknesses of 0.50 and 1.50 mm at maximum pressure due to decreasing temperature at the flow front. In addition, the thin-walled spiral part mold can be filled for a wall thickness of 2.70 and 3.00 mm. In the correlation study conducted for these values, an agreement of approximately 90% was achieved. However, it was also observed that as the pressure increases, the deviation between the experimental and theoretical results becomes more pronounced.

## 1. Introduction

In recent years, the automotive industry has been demanding high-performance [1,2], lightweight [3,4] and low-cost materials [5] to reduce the effects of global warming and carbon emissions [6]. Polymer and polymer composite materials draw attention in terms of developing lightweight products compared to metals [7,8]. Especially, the widespread fabrication of electric vehicles, which promotes polymeric materials used in the automotive industry, becomes even more vital [9]. The production of injection-molded thin-walled plastic parts, which are effective in weight reduction applications, is increasing rapidly [10,11]. The thin-walled plastic parts are affected by the deformations during the injection molding operation [12,13]. The efficiency and properties of the injection molded parts depend on processing parameters such as cooling time, packaging pressure and injection temperature [14,15]. Wang & Cai [16] reported the injection mold design of a notebook battery cover and optimization of the injection processing parameters with Moldflow software. During the injection molding of the thin-walled plastic parts, a reduction of approximately 69% was observed in the deformations. In addition, the quality of injection molded plastic parts depends on the properties of the material, design of parts and mold [17,18,19]. Azad et al. [20] reported that the packing time and melt temperature are the most significant parameters on warpage of high-density polyethylene/recycled polyethylene terephthalate/wood composites. In addition, wood content was found to be the most significant parameter affecting volumetric shrinkage of the composite. Cheng et al. [21] reported that the warpage reduction in the thin-walled parts was dependent on part design, cooling system and molding parameters. Oktem et al. [22] studied the effect of cooling time on the deformation of thin-walled parts by the simulation method.

Optimization of injection molding processing parameters [23,24] was performed by using various methods such as Moldflow simulation [25,26], design of experiments methods (DoEs) and Taguchi experimental design for the quality of the product, performance of the process, as well as material and cost efficiency [27,28]. Song et al. [29] reported that the orthogonal experimental design and the responsive surface experimental design were applied to reduce warpage and volume shrinkage of thin-walled parts. Especially, the DoE is effective for costly injection parameter settings compared to the trial-and-error method. Chen et al. [30] reported the quality in length, warpage, as well as reduction in the costs and time spent on the injection molding by a DoE. Zhiguo et al. [31] reported converting material data from the Moldflow software to Abaqus, which is the structure simulation software used to design thin-walled fiber-reinforced thermoplastic parts. Moayyedian et al. [32] reported that the Taguchi experimental design was applied to minimize the trial numbers and warpage, volume shrinkage and short shot for the quality of the thin-walled plastic part. Azaman et al. [23] simulated the cavity residual stress formation in the shallow thin-walled lignocellulosic polymer composites produced during the injection molding by Moldflow software. Identifying and minimizing possible problems to be encountered in the injection molding of plastic parts with simulations before the experiment also leads to a reduction in the carbon footprint [33,34]. The optimization of thin-walled plastic parts is very effective in increasing sustainability in the automotive industry [35,36]. The automotive industry attaches importance to Moldflow analysis to obtain foresight in minimizing the defects and problems to be encountered in the design and process phase [37]. As a result of the analysis, much data is obtained in terms of part design, mold design and processing parameters [38]. Moldflow software is a simulation program that is useful for the simulation of plastic mold flow and enhances mold design, enabling the production of good-quality products [39]. In Moldflow analysis, preliminary data are obtained for the determination of the runner location in mold production and design, and then, the correct processing parameters and the situations that will be encountered after the part is molded are determined [40,41].

In this study, we aimed to reduce the weight of talc-filled polypropylene (PP) components, widely used in the automotive industry, by designing and employing a thin-walled spiral mold during injection molding. The thin-walled polypropylene spiral parts were determined by theoretical and experimental studies. Moldflow analysis of thin-walled spiral parts was carried out for four thickness values. The thin-walled spiral parts were fabricated by injection molding with a wall-thickness value of 2.70 mm, and different pressure values were applied. Optimum flow length and weight of the thin-walled spiral parts were calculated with theoretical results and experimental measurements.

## 2. Materials and Methods

### 2.1. Material

The 10% talc-filled (by weight) PP copolymer granules with the trade name Hostacom CR 1171 G1 were supplied by LyondellBasell Industries Holdings (Istanbul Office, Turkiye), which is an injection molding grade and long-term ultraviolet radiation (UV) resistance. The components manufactured from 10% talc-filled PP copolymer are the deflector, the bumper and both the exterior and interior trim parts. The Technical Data Sheet (TDS) was provided by the supplier of the PP granules. The properties of PP are melting flow rate (MFR) value is 11 g/10 min (at 230 °C and under 2.60 kg load) according to ISO 1133 [42], the density value is 0.99 g/cm^3^ according to ISO 1183 (Method A), the elastic modulus in the parallel direction is 1614 MPa and the elastic modulus in the vertical direction is 1480 MPa.

### 2.2. Theoretical Methodology

**Figure 1:**
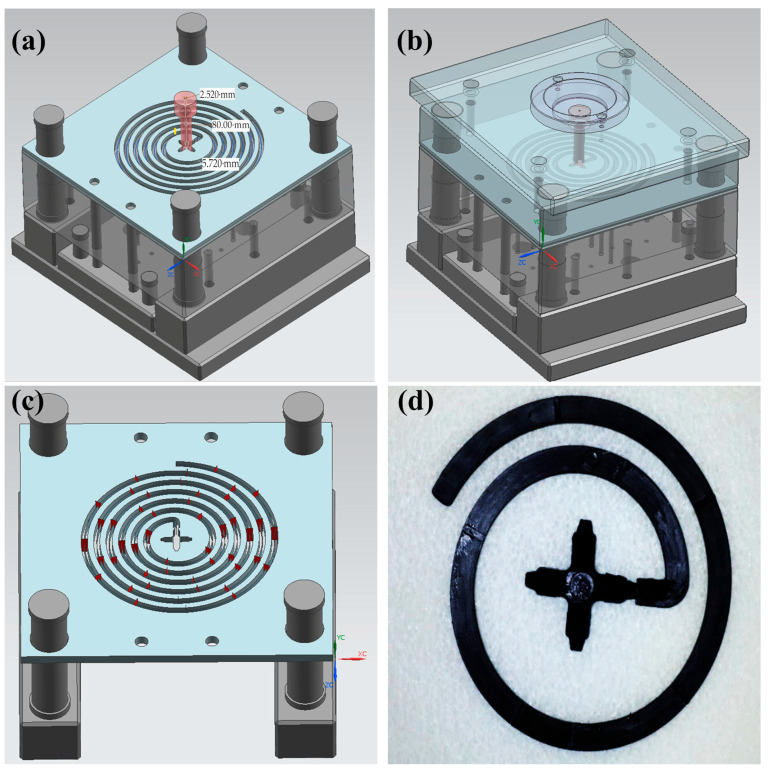
(**a**) Thin-walled spiral part mold sprue gate; (**b**) 3D thin-walled spiral part mold; (**c**) thin-walled spiral part mold separated at 50 mm length; and (**d**) thin-walled spiral part separated at 50 mm length.

Moldflow fully simulates the injection molding process. In injection molding, the flow length decreases in thin-walled parts because the outer walls cool rapidly, which leads to short-shot problems. Therefore, it allows potential errors in part and mold design to be identified before the mold steel is cut. A conventional cold-runner injection mold consisting of upper and lower plates was assumed, and a spiral-shaped part located at the center was modeled together with the gate–sprue–runner system with a fully 3D geometry. The sprue diameter was approximately 2.520 mm, and the runner outer diameter was about 5.720 mm (Figure 1a). A central gating strategy was selected for the spiral geometry, allowing the material to flow uniformly from the center outward. Moldflow analysis reduced the cost and improved productivity of thin-walled automotive plastic parts. Autodesk MoldFlow Insight^®^ 2016 software was used for simulation for the determination of flow length and weight of the spiral parts and mold design. A single-point gate thin-walled spiral part mold with a cold runner is designed to be used in injection molding. The designed spiral part mold is given in Figure 1. 2738 steel was used as the injection mold material to produce the spiral part mold. The mold surface has been completed as finishing, and no surface polishing has been applied. For easy determination, the flow length measuring points are defined on the spiral section with a depth of 0.25 mm and a length of 1.60 mm at 50 mm intervals. The theoretical results were given by the Moldflow simulation. The Moldflow analyses were performed for spiral parts with wall-thickness values of 0.50, 1.50, 2.70 and 3.00 mm at pressure values of 20, 30, 40, 50, 60, 70, 80, 90, 100, 110, 120, 140 and 180 MPa. The one gate was used for the polymer melting filling in the mold cavity, which is located at the central point of the mold. The resulting filling time, clamping force, filling rate, temperature at the flow front, weight and flow length of the spiral parts were analyzed.

### 2.3. Experimental Methodology

**Figure 2:**
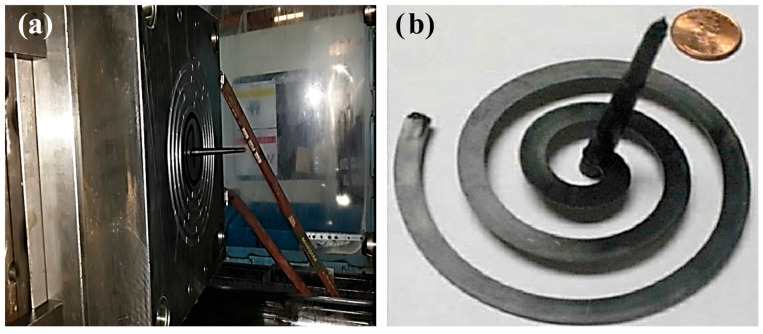
(**a**) The injection thin-walled spiral part mold and (**b**) a fabricated thin-walled spiral part by injection molding.

**Figure 3:**
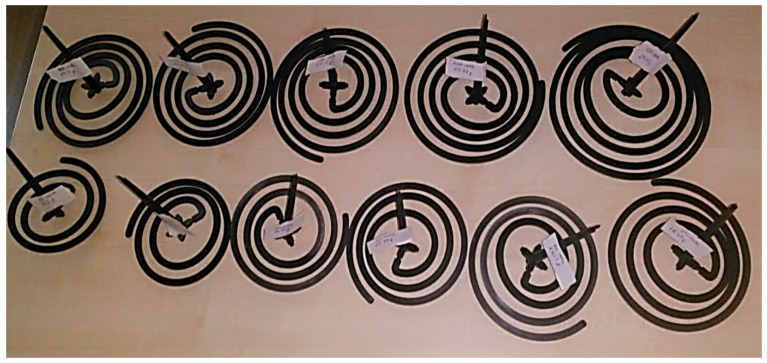
The fabricated thin-walled spiral parts with a wall-thickness value of 2.70 mm at different pressures.

The thin-walled spiral part with a wall-thickness value of 2.70 mm was fabricated by an injection molding for experimental measurements. The thin-walled spiral parts were fabricated by injection molding using an Arburg brand 470C (Arburg GmbH + Co KG, Lossburg, Germany) model injection molding machine with 1500 kN clamping force. The specifications of the injection machine are given in Table 1. The injection thin-walled spiral part mold and the fabricated thin-walled spiral part by injection molding are given in Figure 2. Injection molding was carried out with a mold surface temperature of 40 °C and a melt temperature of 240 °C. The thin-walled spiral parts were fabricated with a wall-thickness value of 2.70 mm at different pressure values. For optimal filling reliability, the simulation was conducted using the process parameters prescribed by Autodesk MoldFlow Insight^®^. During the fabrication of all thin-walled spiral parts, the injection molding parameters such as melt temperature, mold surface temperature and screw speed were kept constant. Also, the injection molding parameters are given in Table 2. The properties of thin-walled spiral parts are given in Table 3. Fabricated thin-walled spiral parts with a wall-thickness value of 2.70 mm at different pressures are given in Figure 3.

## 3. Results

### 3.1. The Filling Time and Filling Rate

**Figure 4:**
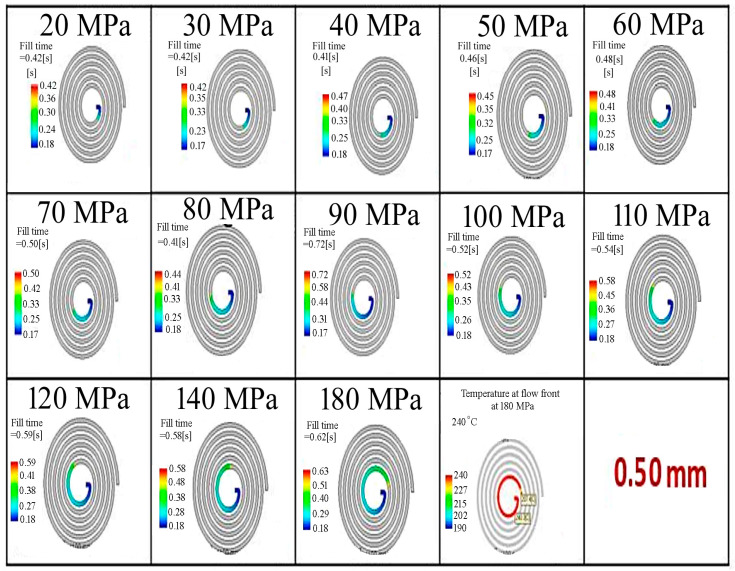
The filling behavior of the thin-walled spiral parts with a wall-thickness value of 0.50 mm according to pressure.

**Figure 5:**
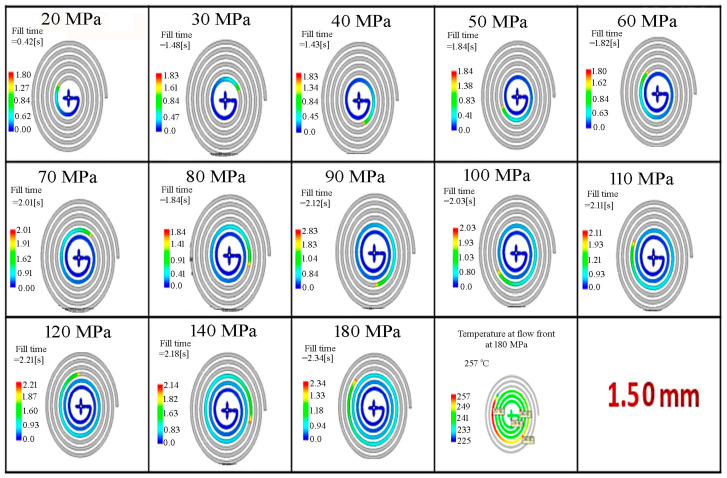
The filling behavior of the thin-walled spiral parts with a wall-thickness value of 1.50 mm according to pressure.

**Figure 6:**
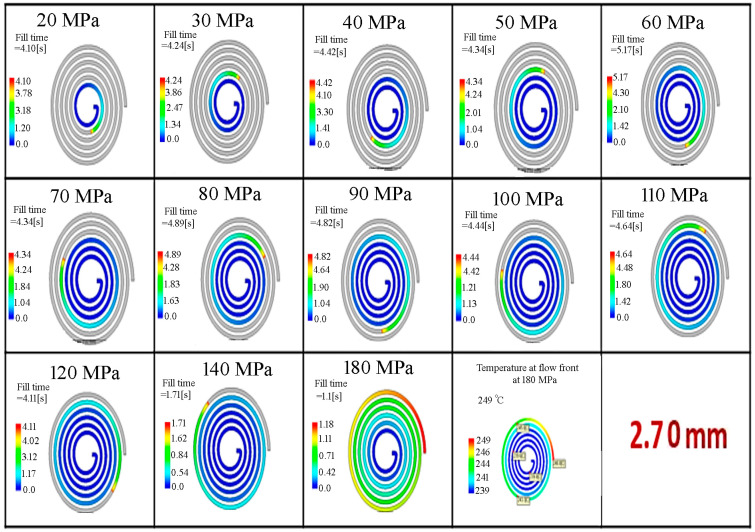
The filling behavior of the thin-walled spiral parts with a wall-thickness value of 2.70 mm according to pressure.

**Figure 7:**
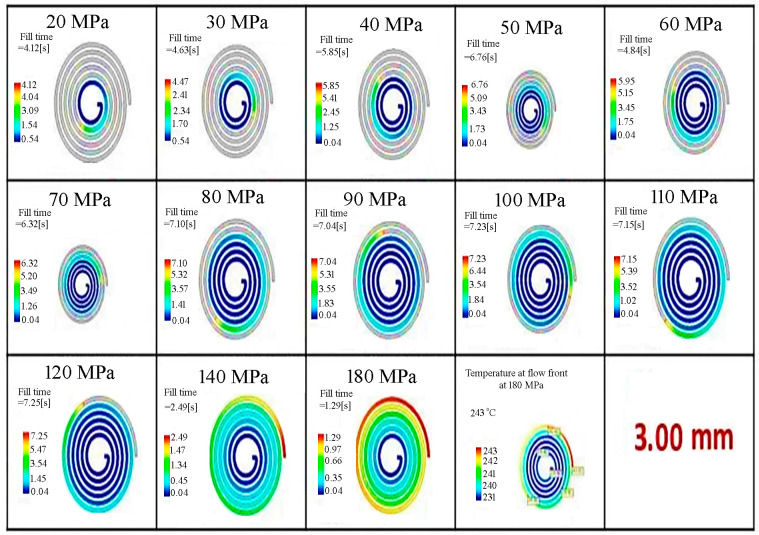
The filling behavior of the thin-walled spiral parts with a wall-thickness value of 3.00 mm according to pressure.

In Moldflow simulations, the filling phase can simulate short-shot risks in thin-walled parts where rapid cooling occurs. A color scale from blue to red is used to indicate the filling sequence. On this scale, blue represents the areas first reached by the molten plastic, while red represents the areas reached last. The filling behavior was determined for the thin-walled spiral parts with wall-thickness values of 0.50, 1.50, 2.70 and 3.00 mm by Moldflow analysis, and is given in Figure 4, Figure 5, Figure 6 and Figure 7, respectively. It is seen that the filling time of the thin-walled spiral part mold is 0.43 s for a wall-thickness value of 0.50 mm, 1.61 s for a wall-thickness value of 1.50 mm, 5.03 s for a wall-thickness value of 2.70 mm and 6.13 s for a wall-thickness value of 3.00 mm at a pressure value of 20 MPa. For the thin-walled spiral part with a wall-thickness value of 0.50 mm, the flow was finished at 0.43 s due to early solidification and low pressure during the injection molding process. However, the filling time for wall-thickness values of 1.50, 2.70 and 3.00 mm increased because the flow was slowed before the front of the flow solidified. It is seen that the mold flow should be slowed down, and the filling time of the thin-walled spiral part mold should be increased. The results indicated that pressure and wall thickness are the most effective factors on the filling time [15]. It showed that the injection molding processing parameters can be adjusted, and the quality of the thin-walled spiral product can be improved.

**Figure 8:**
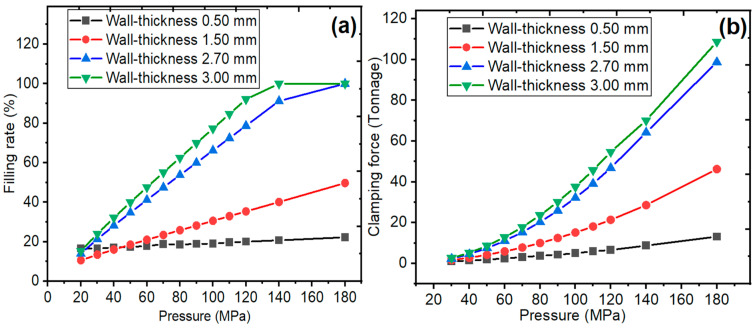
(**a**) The filling rate and (**b**) clamping force of the thin-walled spiral parts according to pressure.

The filling rate values of the thin-walled spiral part mold according to pressure and wall thickness are given in Figure 8a, respectively. During injection molding in molds, the pressure at the machine nozzle changes compared to the pressure on the part at the end of filling, and the difference between them is considered a pressure drop. Pressure decreases during the packing phase for the spiral part with a wall thickness of 0.50 mm at 20 MPa. Afterwards, the pressure and filling rates were fixed. Moldflow analysis showed that the filling rate increased for a thin-walled spiral part with a wall-thickness value of 0.50 mm at a pressure value of 120 MPa. The filling rate was fixed with the decreasing temperature at the flow front. Therefore, the Moldflow analysis resulted in a short shot defect. The flow velocity is the fastest at the runner inlet, and it is seen that the velocity decreases as it moves away from the inlet. The flow front velocity increased, which caused the flow front to easily fill the mold cavity, with an increase in the filling rate [43]. At a thin-walled spiral part with a wall-thickness value of 1.50 mm, the pressure quickly reached a value of 20 MPa. Although the wall-thickness value has increased, the pressure value of 20 MPa is found to be not the ideal value. Reaching the pressure to fill the thin-walled spiral part mold design requires a clamping force. The mold thin-walled spiral part was filled at a pressure value of 180 MPa with a wall-thickness value of 2.70 mm. At the same time, the flow velocity decreased with the decrease in pressure.

### 3.2. The Clamping Force and Temperature at the Flow Front

The clamping force values of the thin-walled spiral part mold according to pressure and wall thickness are given in Figure 8b, respectively. The clamping force was increased as a function of pressure and wall thickness. The clamping force values increased with increasing pressure during the injection molding process. In addition, the clamping force values changed depending on the pressure for all wall-thickness values. The clamping force increased with the increasing wall thickness at all pressures. The maximum clamping force value is 98.74 tonnage for the thin-walled spiral part with a wall-thickness value of 2.70 mm. The clamping force values changed from 2.32 to 98.74 tonnage. It is shown that the increasing wall thickness needs to increase the injection molding capacity in terms of clamping force. It could increase the cost of the injection molding process [44].

**Figure 9:**
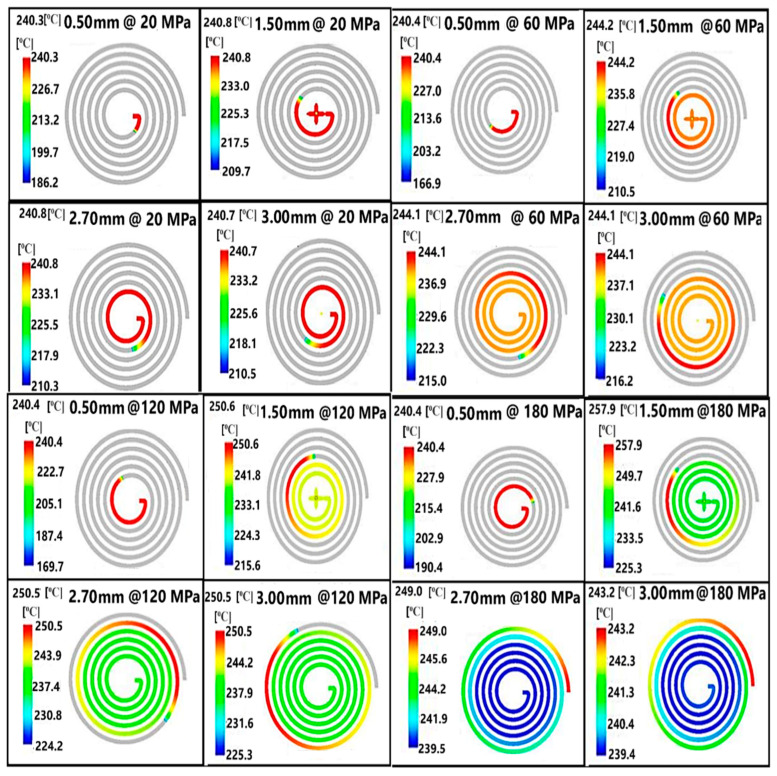
The temperature at the flow front values of the thin-walled spiral parts according to wall thickness at pressure values of 20, 60, 120 and 180 MPa.

The data obtained from the Moldflow analysis illustrates the distribution of flow front temperature variations during the filling phase, aimed at preventing material degradation and surface defects. In the color scale, red corresponds to regions of highest temperature, whereas blue represents regions of lowest temperature. Temperature at the flow front was determined for the thin-walled spiral parts with wall-thickness values of 0.50, 1.50, 2.70 and 3.00 mm by Moldflow analysis according to pressure. The temperature at flow front values of the thin-walled spiral parts according to wall-thickness at pressure values of 20, 60, 120 and 180 MPa are given in Figure 9. The temperature at the flow front increased depending on the thickness of the thin-walled spiral parts at the same pressure value. When the temperature at the flow front increases with flow length from the runner, the polymer is exposed to an increasing friction and shear rate. The temperature at the flow front was 240.80 °C at a pressure value of 20 MPa for a wall-thickness value of 2.70 mm. It was found that a pressure value of 20 MPa was not sufficient for filling the thin-walled spiral parts mold for all wall-thickness values. The flow front solidifies later with the increase of the flow length. It is seen that the flow length increased with increasing pressure. The temperature at the flow front was 244.10 °C at a pressure value of 60 MPa. The temperature at the flow front was 250.50 and 249.00 °C at a pressure value of 120 and 180 MPa, respectively. The temperature at the flow front increases as the flow length increases. When the pressure is not enough to fill the thin-walled spiral part mold, the temperature at the flow front drops and solidifies. Due to the high injection speed at pressure values of 120 and 180 MPa, the temperature of the front flow increased with the increasing friction rate. This can cause degradation or combustion of the material at high pressure.

### 3.3. Weight and Flow Length

**Figure 10:**
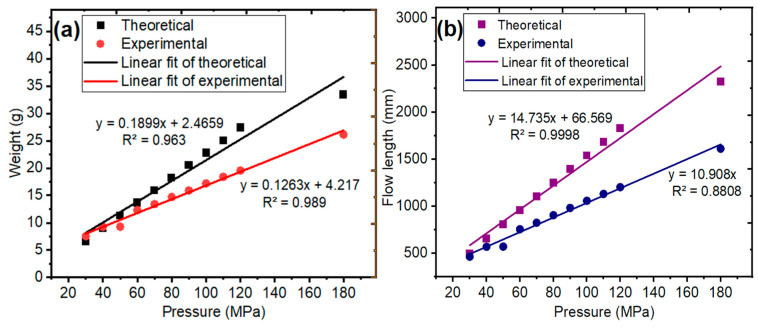
The correlation graph of theoretical and experimental (**a**) weight, as well as for the (**b**) flow length for a wall-thickness of 2.70 mm, thin-walled spiral part.

The theoretical and experimental weight and flow length values of the thin-walled spiral parts with a wall-thickness value of 2.70 mm according to pressure are given in Figure 10. The weight of the thin-walled spiral parts correlation graph was obtained from simulation results and experimental measurements. The weight correlation graph of the thin-walled spiral parts according to pressure is given in Figure 10a. The weight of the thin-walled spiral part was increased as a function of pressure. The experimental and theoretical *R*^2^, which is the correlation coefficient value for the weight of thin-walled spiral parts, was obtained to be above 0.90. It was shown that there is a good relationship between theoretical and experimental weight data of the thin-walled spiral part since they are close to 1.00. Moldflow simulations are good supportive methods for experimental measurements [45]. Cao et al. [46] determined the mold structure and material for a thin-walled plastic part, which was an automobile audio shell. Determination of the optimizing parameters of the injection molding process is the most economical and effective method to manufacture the products.

The effect of wall thickness on the mold flow behavior of the thin-walled spiral parts was investigated with respect to pressure. The flow front velocity and the flow length values of the thin-walled spiral parts increase proportionally with the effect of pressure at the same thickness. The flow length in all thin-walled spiral parts was filled at a pressure value of 140 MPa for a wall-thickness value of 3.00 mm. In addition, the flow length values of the thin-walled spiral parts increased with the increasing wall thickness. In addition, solidification is prevented along the flow length. Also, the pressure is low in the region where the wall-thickness of the thin-walled spiral parts is high; the obtained flow length could not be achieved at a pressure value of 180 MPa. Moldflow results show that a thin-walled spiral part with a wall-thickness value of 2.70 mm has a weight of 33.43 g and a flow length of 2323.62 mm at a pressure value of 180 MPa. However, its experimental measurements show that the thin-walled spiral part has a weight of 26.11 g and a flow length of 1611.51 mm. The correlation graph of flow length was obtained from data in simulation results and experimental measurements. The flow length correlation graph of the thin-walled spiral parts according to pressure is given in Figure 10b. The flow length of the thin-walled spiral part was increased as a function of pressure. The experimental and theoretical *R*^2^, which is the correlation coefficient value for the weight of thin-walled spiral parts, was obtained to be above 0.90. It was shown that there is a good relationship between theoretical and experimental flow length since they are closer to 1.00. Using Moldflow simulations to investigate the injection molding process parameters for thin-walled spiral parts yielded good agreement between the experimental results and the predicted flow behavior [47,48]. As reported in previous studies [49], improvements in part quality and reductions in manufacturing costs are associated with comprehensive process optimization. Optimum pressure ensures complete mold filling while minimizing sag marks and voids, improving dimensional accuracy and surface finish, thereby enhancing overall part quality. At the same time, it limits energy consumption and reduces wear on the mold and machine, contributing to lower manufacturing costs. It is observed that the simulation results are like experimental measurements [50,51].

## 4. Conclusions

In this study, the optimization of processing parameters of injection-molded thin-walled spiral parts for the automotive industry was investigated by theoretical modelling and experimental studies. It was determined that the pressure and the wall-thickness values had a great effect on the filling of the thin-walled spiral part mold. The filling time of the thin-walled spiral parts is shortened by the effect of high pressure. The clamping force values increased with the increase in pressure values in the thin-walled spiral parts with the same wall-thickness values. The wall-thickness values of the thin-walled spiral parts increased with the increase in the flow length values. Due to the late solidification of the flow front and the effect of pressure, the thin-walled spiral parts with a wall-thickness value of 3.00 mm could be filled at a pressure value of 140 MPa. The thin-walled spiral part with a wall thickness of 2.70 mm was filled at a pressure of 180 MPa. After the pressure reached its maximum, it dropped to 80%, causing a decrease in flow rate while the filling percentage and part weight increased with pressure. It was confirmed that the level of each pressure value to minimize the injection process was reasonable in performing the thin-walled spiral part with a wall thickness value of 2.70 mm, and the flow length and weight results were also confirmed. Finally, the correlation of theoretical results and experimental measurements indicated that Moldflow simulation can be an effective optimization of injection molding parameters for the thin-walled plastic parts. For the injection molding process, the flow parameters of the spiral plastic part were analyzed using Moldflow software, and the results were found to be applicable for the real-time manufacturing process of thin-walled automotive parts.

## Figures and Tables

**Table 1:** Injection molding machine specifications.

Machine Specifications	Value	Unit
Screw diameter	45	mm
Effective screw length (L/D)	18	---
Screw length	160	mm
Measurement screw volume	254	cm^3^
Press capacity	232	g
Shot capacity	35	kg/h
Injection pressure	1580	bar
Packing pressure	1580	bar

**Table 2:** Injection molding processing parameters.

Parameters	Value	Unit
Melt temperature	234–212–201–105	°C
Mold surface temperature	40	°C
Screw rate	35	mm/s
Injection pressure (V/P)	30–120	MPa
Cooling time	20	s

**Table 3:** Properties of thin-walled spiral part.

Properties	Value	Unit	Flow Length
Total volume (t = 1.50 mm)	21.09	cm^3^	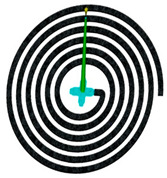
Total length	2323.62	mm	
Width	6.00	mm	
Wall thickness	1.50	mm	
Surface area	13,941.70	mm^2^	

The 2D drawing in Table 3 illustrates the spiral part in black, the gate in blue, and the runner in green.

## Data Availability

The original contributions presented in this study are included in the article. Further inquiries can be directed to the corresponding author.
